# Highly selective SGLT2 inhibitor dapagliflozin reduces seizure activity in pentylenetetrazol-induced murine model of epilepsy

**DOI:** 10.1186/s12883-018-1086-4

**Published:** 2018-06-07

**Authors:** Mumin Alper Erdogan, Dimas Yusuf, Joanna Christy, Volkan Solmaz, Arife Erdogan, Emin Taskiran, Oytun Erbas

**Affiliations:** 1 Department of Physiology, Faculty of Medicine, Izmir Katip Celebi University, Izmir, Turkey; 2 Faculty of Medicine, University of Alberta, Edmonton, AB Canada; 3 Simon Fraser University, Burnaby, BC Canada; 4 Department of Neurology, Faculty of Medicine, Trakya University, Edirne, Turkey; 5 Department of Emergency Medicine, Izmir Bozyaka Training and Research Hospital, Izmir, Turkey; 6 Department of Internal Medicine, Tepecik Training and Research Hospital, Izmir, Turkey; 7 Department of Physiology, Faculty of Medicine, Bilim University, Istanbul, Turkey

**Keywords:** Dapagliflozin, Epilepsy, Pentylenetetrazol, Sodium-glucose linked transporter

## Abstract

**Background:**

Worldwide, over 10 million individuals suffer from drug-resistant epilepsy. New therapeutic strategies are needed to address this debilitating disease. Inhibition of sodium-glucose linked transporters (SGLTs), which are variably expressed in the brain, has been demonstrated to reduce seizure activity in murine models of epilepsy. Here we investigated the effects of dapagliflozin, a highly competitive SGLT2 inhibitor currently used as a drug for diabetes mellitus, on seizure activity in rats with pentylenetetrazol (PTZ) induced seizures.

**Methods:**

Laboratory rats (*n* = 48) were evenly randomized into two experiments, each with four study arms: (1) a vehicle-treated (placebo) arm infused with saline; (2) a control arm infused with PTZ; (3) a treatment arm with PTZ and dapagliflozin at 75 mg/kg, and (4) another treatment arm with PTZ and dapagliflozin at 150 mg/kg. Study subjects were assessed for seizures either via EEG as measured by spike wave percentage (SWP), or clinically via Racine’s scales scores (RSS) and time to first myoclonic jerk (TFMJ).

**Results:**

Rats treated with dapagliflozin had lower mean SWP on EEG (20.4% versus 75.3% for untreated rats). Behaviorally, treatment with dapagliflozin improved means RSS (2.33 versus 5.5) and mean TFMJ (68.3 versus 196.7 s). All of these findings were statistically significant with *p*-values of < 0.0001. There was a trend towards even better seizure control with the higher dose of dapagliflozin at 150 mg/kg, however this was not consistently statistically significant.

**Conclusions:**

Dapagliflozin decreased seizure activity in rats with PTZ–induced seizures. This may be explained by the anti-seizure effects of decreased glucose availability and a reduction in sodium transport across neuronal membranes which can confer a stabilizing effect against excitability and unwanted depolarization. The potential clinical role of dapagliflozin and other SGLT2 inhibitors as anti-seizure medications should be further explored.

## Highlights

- More effective treatments are needed for drug–resistant epilepsy
- Dapagliflozin, a potent SGLT2 inhibitor used for diabetes, may have anti-seizure effects
- Rats with PTZ–induced seizures had abatement of seizure activity when exposed to dapagliflozin
- The potential role of dapagliflozin as an antiepileptic drug should be further explored

## Background

### Epidemiology of drug-resistant epilepsy

Worldwide, over 50 million people live with epilepsy, and about 10 million develop drug-resistant epilepsy, an often-debilitating disease. It is estimated that approximately 2 million people in the United States have epilepsy [1]. Despite ongoing developments in the diagnosis and management of epilepsy, about 20 to 30% of patients have epilepsy that is resistant to treatment, which is often called drug-resistant epilepsy. Commonly used antiepileptic drugs (AEDs) can have pronounced side effect profiles, low tolerability, and narrow therapeutic ranges. Because of this, patients who require multiple AEDs to control their epilepsy often experience adverse effects that can range from somnolence, headaches, blunting of cognition, to more serious reactions that include mood changes, arrhythmias, and interference with the functioning of other drugs. Despite numerous studies to advance the treatment of epilepsy, the ideal treatment remains elusive for many patients who live with refractory disease [2].

### Intracerebral transport of glucose

Glucose is the primary metabolic substrate for the brain, and a continuous supply of glucose is required for normal neuronal function [3]. Glucose is transported into brain across the blood-brain barrier (BBB) and made available to neurons and glial cells via specific transporters. Glucose transporters (GLUTs) exist in two major groups: [1] the GLUT energy-independent facilitated transporters [4]; and [2] the SGLT sodium-glucose linked transporters. With regards to the first group, GLUT1 is expressed in the BBB and glial cells, while GLUT3 is expressed in neurons [5]. With regards to the SGLTs, these transporters were first identified in the intestine and kidney [6, 7]. We are only beginning to understand the physiological roles and importance of SGLTs in the brain. Recently, it was revealed that SGLTs are widely expressed in the brain, especially in the cerebellum, hippocampus, frontal cortex, caudate nucleus, putamen, amygdala, parietal cortex, and paraventricular nucleus of the hypothalamus. SGLTs, however, appear to be poorly expressed in the brain stem [8]. Considering widespread expression of SGLTs in neuronal tissue, we are interested in further investigating the potential roles that SGLTs may have in the development of neuropathological conditions such as epilepsy, as well as how the expression or activity SGLTs can modified to treat these conditions.

### Attenuating seizure activity via inhibition of SGLTs

Once existing in obscurity, SGLTs are now a focus of interest in the medical community, after it was discovered that inhibition of SGLT2 can result in improved plasma glucose control for patients living with diabetes mellitus type 2 (DMT2). SGLTs are highly expressed in the kidney, and are responsible for approximately 90% of renal glucose reabsorption in the proximal convoluted tubules [6, 7]. By disabling SGLT2 activity with inhibitors such as dapagliflozin, reabsorption of glucose into the bloodstream can be significantly diminished, leading to glycosuria but avoidance of hyperglycemia.

Over the past 10 years, sodium-glucose cotransporter 2 (SGLT2) inhibitors such as dapagliflozin have become a new and clinically very important class of oral drugs for the treatment of DMT2 [9]. Dapagliflozin inhibits SGLT2 in a competitive, reversible, and highly selectively manner. As per above, dapagliflozin prevents reabsorption of glucose in the proximal convoluted tubules of the kidneys and therefore induces loss of glucose from the body and into the urine [10].

While a number of classes of diabetic drugs such as insulins and sulfonylureas are also effective at lowering blood glucose levels through a variety of different mechanisms, SGLT2 inhibitors are rather unique in that its glucose lowering activity is diminished when serum glucose concentrations are normal. This significantly decreases the risk of hypoglycemia, something that is highly desirable in the clinical practice of managing diabetes.

Furthermore, recent data revealed that SGLT2 inhibitors such as dapagliflozin may have additional ancillary benefits beyond maintaining euglycemia. Subsequent analysis from the CVD-REAL Nordic trial revealed that dapagliflozin may also reduce the risk of hospitalization due to kidney disease, heart failure, and death from any cause in patients with diabetes [11]. For these many reasons, over the past 5 years the number of patients on dapagliflozin and other SGLT2 inhibitors have increased dramatically.

In one recent study [12], Melo et al. demonstrated that phlorizin, a specific SGLT inhibitor, increased the severity of limbic seizures induced by pilocarpine during status epilepticus (SE) in mice, and in addition, caused more extensive neurodegeneration in the hippocampus 24 h after SE. However, yet other studies with the same compound and similar methodologies yielded opposite results. Given early conflicting results surrounding SGLT inhibition and changes to seizure activity and the increasing use of SGLT2 inhibitors among patients with diabetes, as well as the possibility that SGLT2 inhibitors may decrease seizure activity in pre-clinical models and thus may serve as a potential AED for patients with drug-resistant seizures, we were motivated to conduct this study to further investigate the effects of dapagliflozin on seizure activity in the PTZ murine model for epilepsy.

## Methods

### Ethics oversight

Prior to the start of the study, ethics approval was obtained from the appropriate animal research ethics committee that is responsible for overseeing research activity in our university and institution, the Ege University Institutional Animal Care and Ethical Committee. The ethics certificate is available through the corresponding author. All experiments as well as the treatment of laboratory animals were carried out in accordance to the Guide for the Care and Use of Laboratory Animals, as published by the United States National Institutes of Health (NIH).

### Study subjects and location

All experiments were performed in the biomedical research laboratories of the Ege University Faculty of Medicine in İzmir, Turkey. Forty-eight (48) male Sprague–Dawley rats (Charles River) were used in this study, each weighing 200 to 250 g. The rats were purchased from the Saki Yenilli Experimental Animal Production Laboratory AS in Ankara, Turkey. Half of the rats [24] were randomized for EEG-based measurement, and non–placebo (non–vehicle–treated) members of this group were exposed to a lower dose of PTZ at 35 mg per kilogram (mg/kg). The other half of the rats [24] were randomized for behavioral measurement, and non–vehicle–treated members of this group were exposed to a higher dose of PTZ at 70 mg/kg to increase the likelihood of clinically apparent and evaluable seizures. The rats were kept on a 12 h–12-h light–dark cycle with light from 07:00 to 19:00 h in clean, quiet rooms, with an ambient temperature of 22 to 24 degrees Celsius (°C). They were fed standard laboratory food and provided with tap water to drink ad libitum. Euthanization, as applicable, was done humanely by trained laboratory personnel with a high dose of intravenous ketamine (80 mg/kg) followed by cervical dislocation.

### Drugs and dosing

Purified, research grade PTZ and dapagliflozin were obtained from reputable sources and dissolved in 0.9% saline. Drug solutions were prepared freshly in the morning on the days infusions took place. Rats in the EEG arm received either saline (placebo), 75 mg/kg or 150 mg/kg of dapagliflozin intraperitoneally (IP), and 35 mg/kg of PTZ IP. Rats in the clinical observation arm received either saline (placebo), 75 mg/kg or 150 mg/kg of dapagliflozin IP, and 70 mg/kg of PTZ IP.

Dapagliflozin at 75 and 150 mg/kg IP would be supratherapeutic for a human subject (for humans, dapagliflozin is typically dosed at 5 mg to 10 mg PO daily or about 0.1 mg/kg), but these doses are necessary in rats to account for their faster drug metabolism, which can easily exceed that of a human by a multiple of ≥10. The dosing of PTZ for murine epilepsy research has been well studied and in this study we followed established conventions of PTZ dosing. The PTZ dose of 35 mg/kg IP is ideal for inducing epileptiform activity that are adequately observed on EEG. However, this dose is too small to consistently produce clinically-apparent seizures. Conversely, the PTZ dose of 70 mg/kg IP is adequate to elicit clinically-apparent seizures, but it is not ideal for EEG studies as it tends to result in increased noise and therefore narrower signal to noise ratio.

### Randomization and blinding

Forty-eight rats were randomized into two groups: 24 rats for EEG measurement (Group A), and 24 rats for behavioral measurement (Group B). The rats were randomized purely and electronically without stratification, and investigators who were responsible for evaluating the rats’ clinical activity and EEG data were made not aware of which intervention arm each rat belonged to.

### Experiment involving EEG measurement

For the EEG group, we installed intracranial electrodes in each rat to facilitate measurement. The rats were deeply anesthetized during this procedure. Following adequate anesthetization, a small cephalic hole was created stereotaxically with a sterile drill. Electrode wires for EEG recording—polyamide-coated stainless steel wires that measured 0.1 mm in diameter and with an electrical resistance of less than 1 Ω per 10 mm—were implanted on the dura over the left frontal cortex 2.0 mm lateral to the midline, 1.5 mm anterior to the bregma and a reference electrode was implanted over the cerebellum 1.5 mm posterior to the lambda, along the midline [13, 14]. These electrodes were fixed into place with dental acrylic, a mixture of numerous resins and alloys used for dental restoration. Twelve days after implantation of the electrodes, the 24 rats in Group A were sedated with ketamine at 80 mg/kg and xylazine 4 mg/kg IP, and further randomized into 4 arms (each arm *n* = 6) designated A1, A2, A3, and A4.

A1 was a control arm and received only saline placebo. A2 was also administered saline IP but will later be given PTZ. A3 was administered saline with dapagliflozin at 75 mg/kg IP and A4 was administered saline with dapagliflozin at 150 mg/kg IP. About 30 min after dapagliflozin administration, all arms except A1 were given PTZ at 35 mg/kg IP. EEG recording began 5 min after PTZ administration, while the rats were sedated, awake, and kept in a special container.

All EEG recordings and behavioral assessment protocols were performed as previously described [15]. In summary, recording of EEG activity was done via a BIOPAC MP150 Data Acquisition System (by BIOPAC System Incorporated, Santa Barbara, CA, USA) and continued for 60 min. The signals were amplified 10,000 times and filtered through a range of 1–60 Hz (Hz).

To determine the onset of seizure activity on the EEG, trained neurophysiologists review the tracing for spike waves, which is objectively defined as the presence of a high amplitude wave that is at least twice as high as the baseline values. We utilized spike wave percentage as our endpoint since this is a familiar and reproducible method for quantifying epileptiform activity in murine experiements [16]. There are several steps involved in obtaining the spike wave percentage. First, continuous EEG data is split into 1–second bins. Each bin is analyzed by clinical neurophysiologists for the presence of epileptiform spike waves. As previously stated, the onset and cessation of a spike wave complex is defined by the presence of a high amplitude wave that is at least twice as high as the baseline values. If there is at least one spike wave within the bin, the bin is deemed positive. If there are no spike waves within the bin, the bin is deemed negative for spike waves. The overall “spike wave percentage” is derived by dividing the number of positive bins by the total number of bins in the EEG data [15]. This metric is calculated after every 2 min of continuous EEG data. In our study, two clinical neurophysiologists, who are blinded to which arm the data belongs to, independently reviewed the EEG waveforms to look for spike waves and calculate the spike wave percentages.

### Experiment involving behavioral measurement

Rats randomized to Group B—the behavioral measurement group—were further randomized into 4 treatment arms B1, B2, B3 and B4, with *n* = 6 in each arm. The first arm (B1) was a control arm and only received IP saline. Arm B2 received IP saline as placebo, and subsequently received IP PTZ for the induction of seizure activity. Arm B3 received dapagliflozin at 75 mg/kg IP. Arm B4 received dapagliflozin at 150 mg/kg IP. About 30 min after infusion of dapagliflozin, all arms except B1 received PTZ 70 mg/kg IP.

The rats were individually observed for seizure activity with Racine’s scale for PTZ-induced seizures in rats and also time to first myoclonic jerk (TFMJ) as previously described [15]. Scores for Racine’s scale were recorded as: 0 for no convulsion; 1 for twitching of vibrissae and pinnae; 2 for motor arrest with more pronounced twitching; 3 for motor arrest with generalized myoclonic jerks (*n.b.* this event would also mark TFMJ); 4 for tonic–clonic seizure while the animal remained on its feet; 5 for tonic–clonic seizure with loss of the righting reflex; and 6 for lethal seizure. The onset times were recorded in seconds. Almost all animals that developed tonic generalized extension died. The observation period for PTZ-induced seizures were limited to 30 min [15]. After this duration, the animals were euthanized.

### Statistical analysis

Results were expressed as a mean ± standard deviation (SD) from the mean. We used version 15.0 of the SPSS software package for Windows to facilitate statistical analysis. We also used the Shapiro-Wilk test to determine if the population of values has a normal distribution. Racine’s scale scores were evaluated with the Kruskal–Wallis one–way analysis of variance test and, and TFMJ were evaluated by one-way analysis of variance (ANOVA). The post-hoc Bonferroni and Mann Whitney U test were utilized to identify differences between the experimental groups. The value of *p* < 0.05 was accepted as statistically significant.

## Results

Across both the EEG and behavioral groups, the addition of dapagliflozin resulted in markedly decreased seizure activity as measured via EEG SPW, Racine’s scale scores, and TFMJ.

### EEG spike wave percentage

**Fig. 1:**
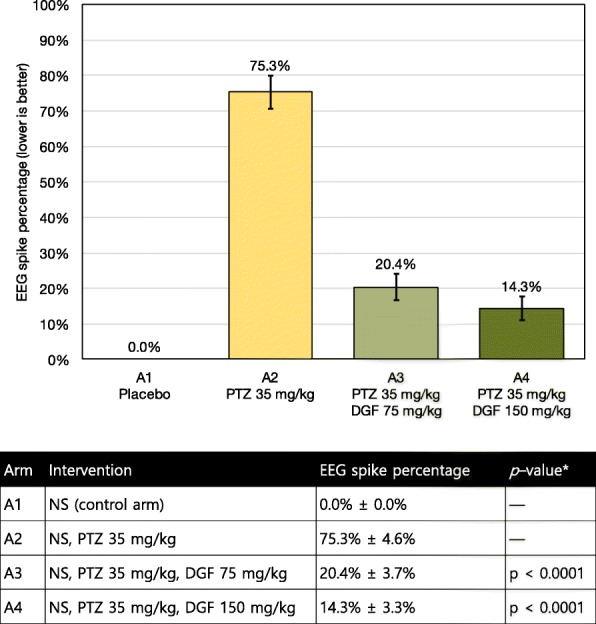
Effect of dapagliflozin on epileptiform activity on EEG as measured by spike percentage for PTZ–induced seizures in rats. When compared to the A2 arm in which no dapagliflozin (DGF) was administered, intraperitoneal (IP) dapagliflozin at 75 mg/kg and 150 mg/kg were able to significantly reduce mean EEG spike percentage by over 3 times, with *p*–value of < 0.0001, in rats treated with 35 mg/kg of IP pentylenetetrazol (PTZ). The ± range represents the standard deviation (SD) from the mean

**Fig. 2:**
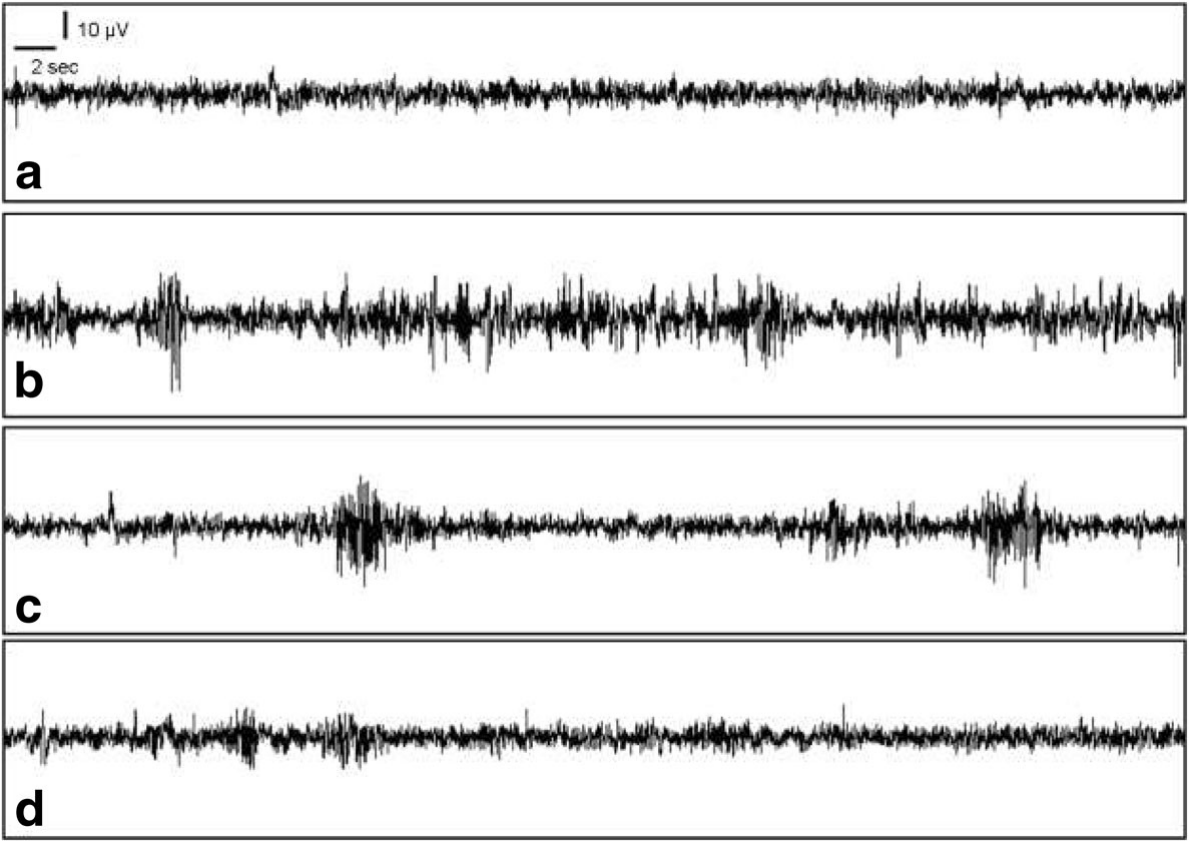
Effect of dapagliflozin on EEG tracing. The addition of dapagliflozin at 75 mg/kg IP (row c) and 150 mg/kg IP (row d) resulted in fewer epileptiform activity as compared to no dapagliflozin (row b) in rats treated with 35 mg/kg of IP pentylenetetrazol (PTZ). Row a is a vehicle-treated (placebo) group that received neither PTZ nor dapagliflozin

The addition of dapagliflozin at 75 mg/kg IP and subsequently 150 mg/kg IP resulted in a statistically significant reduction in epileptiform activity as measured via SPW (see Figs. 1 and 2). Without dapagliflozin, the PTZ-treated A2 arm had a spike wave percentage score of 75.3%. With the administration of dapagliflozin at 75 mg/kg, the A3 arm had a spike wave percentage score of 20.4%, which represents an absolute reduction of 54.9% when compared to the PTZ-only A2 arm. With the administration of dapagliflozin at 150 mg/kg, the A4 arm had a spike wave percentage score of 14.3%, which represents an absolute reduction of 61% when compared to the PTZ-only A2 arm. There was 50% mortality in the A2 arm which was treated with PTZ at 70 mg/kg and saline. There were no mortality in other arms during the EEG experiments.

One-way ANOVA and post hoc Bonferroni test revealed that the difference between A3 and A2, as well as A4 and A2, were both statistically significant with *p*-values of < 0.0001. The difference between the lower and higher doses of dapagliflozin (A3 vs A4) however, was not deemed statistically significant (*p* > 0.05). Additional parameters and metrics from the EEG experiments are presented in Table 1, and recovery times in Table 2.

**Table 1:** Additional parameters and results from the EEG experiments

Arm	Seizure onset (seconds)	Duration of single seizures (seconds)	Duration of high frequency or high amplitude spiking (seconds)	Total seizure duration (seconds)
A1	0 ± 0.0	0 ± 0.0	0 ± 0.0	0 ± 0.0
A2	45.08 ± 9.3	10.2 ± 3.10	5.3 ± 0.93	2564.2 ± 105.6
A3	110.3 ± 16.8**	6.06 ± 1.08*	3.2 ± 0.85*	685.2 ± 60.8**
A4	185.7 ± 12.1**	2.1 ± 0.93*	0.8 ± 0.12*	451.8 ± 35.9**

The treatment arms are as follows: A1 for saline (control arm), A2 for PTZ at 70 mg/kg and saline, A3 for PTZ at 70 mg/kg and dapaglifozin at 75 mg/kg, A4 for PTZ at 70 mg/kg and dapaglifozin at 150 mg/kg. Superscript (*) denotes a statistically significant difference between the current arm and A2, with *p* < 0.001. Superscript (**) denotes a statistically significant difference between the current arm and A2, with *p* < 0.05

**Table 2:** Recovery times from the EEG experiments

Arm	Intervention	Recovery time (seconds)
A1	Saline control arm	0 ± 0.0
A2	PTZ at 70 mg/kg and saline	565.8 ± 12.7
A3	PTZ at 70 mg/kg and dapaglifozin at 75 mg/kg	483.3 ± 35.2*
A4	PTZ at 70 mg/kg and dapaglifozin at 150 mg/kg	368 ± 24.9*

Superscript (*) denotes a statistically significant difference between the current arm and A2, with *p* < 0.05

### Racine’s stages scores

The addition of dapagliflozin significantly improved Racine’s stages scores (RSS). The six vehicle–treated rats in arm B1 had no discernible convulsions. In arm B2, the addition of 70 mg/kg of PTZ IP resulted in high RSS as expected (mean score of 5.5). In arm B3, the addition of dapagliflozin at 75 mg/kg IP resulted lower RSS (2.33 ± 0.5). In arm B4, the addition of dapagliflozin at 150 mg/kg IP also resulted lower RSS (2.1 ± 0.5).

Both the Kruskal–Wallis and Mann–Whitney U tests demonstrated that the differences between B1 versus B2, as well as B2 versus B3 and B2 versus B4 statistically significant with a *p*-value of < 0.0001. The difference in RSS between arms B3 and B4, in which either 75 or 150 mg/kg of IP dapagliflozin were given, was deemed not statistically significant with a *p*–value of > 0.05 (Table 3).

**Table 3:** Effect of dapagliflozin on Racine’s scores and TFMJ for PTZ–induced seizures in rats

Arm	Intervention	Racine’s	TFMJ (s)	*p*–value
B1	NS (control arm)	0 ± 0.0	Not reached	–
B2	NS, PTZ 70 mg/kg	5.5 ± 0.2	68.3 ± 3.4 s	–
B3	NS, PTZ 70 mg/kg, DGF 75 mg/kg	2.33 ± 0.5	196.7 ± 23.0 s	*p* < 0.0001
B4	NS, PTZ 70 mg/kg, DGF 150 mg/kg	2.1 ± 0.5	268.3 ± 23.1 s	*p* < 0.0001

When compared to the B2 no dapagliflozin (DGF) arm, intraperitoneal (IP) dapagliflozin at 75 mg/kg and 150 mg/kg resulted in significantly reduced mean Racine’s scores by over 3 points, and improved mean time to first myoclonic jerk (TFMJ)—as measured in seconds—by a factor of over 2.5×, with *p*–value of < 0.0001, in rats treated with 70 mg/kg of IP pentylenetetrazol (PTZ). The ± range represents the standard deviation (SD) from the mean

### Time to first myoclonic jerk

The addition of dapagliflozin significantly increased the TFMJ. The 6 rats in arm B1 (vehicle-treated rats) had no discernible myoclonic jerks, and therefore the TFMJ was recorded as not reached. IP injection of PTZ at 70 mg/kg in arm B2 resulted in myoclonic jerks with a mean TFMJ of 68.3 s. The addition of dapagliflozin 75 mg/kg IP to PTZ-treated rats in arm B3 significantly increased mean TFMJ to 196.7 s. Rats treated dapagliflozin 150 mg/kg IP in arm B4 had a mean TFMJ of 268.3 s.

One-way ANOVA and post hoc Bonferroni tests found that difference in mean TFMJ between B1 versus B2, B2 versus B3, and B2 versus B4 were all statistically significant with *p*–values of < 0.0001 (Table 3).

## Discussion

In this study, we demonstrated that dapagliflozin, a highly selective and reversible inhibitor of SGLT2, had clinically and electrophysiologically apparent anticonvulsive effects for rats with PTZ–induced seizures. To our knowledge this is the first published study that reported the potential anticonvulsive effects of dapagliflozin.

### Glucose metabolism in the CNS

Neurons require high amounts of energy during normal brain function. The central nervous system (CNS) consumes ten times more energy than the average of other tissues and under ideal conditions it utilizes glucose nearly exclusively as its main energy substrate. The transport of systemic glucose through the BBB and into neuronal as well as glial tissues is a fundamentally essential process, and abnormalities in these pathways are known to cause serious disorders, including several epilepsy syndromes such as GLUT1 deficiency [17], that oftentimes manifests in children.

While glucose transporter 1 (GLUT1) is the predominant isoform expressed in the BBB and glial cells [18], glucose transporter 3 (GLUT3) is the dominant isoform expressed in neurons [19]. Sodium–glucose linked transporters (SGLTs) are transcribed from genes that belong to the solute carrier 5A (SLC5A) family of genes, which harness the gradient of sodium ions across the plasma membrane to drive glucose and galactose into cells [6, 20, 21]. The most studied members of this family are *SGLT1* and *SGLT2*, whose products are involved in glucose transport in specialized regions of the mammalian brain [8, 22]. Since these pioneering studies, it has been established that SGLT1 is expressed in many areas of the brain, including CA1, CA3, and the dentate gyrus hippocampal subfields [23], while significant SGLT2 expression has been detected in the hippocampus and cerebellum.

### Augmenting glucose activity as a seizure control strategy

The augmentation of glucose transport and metabolism, typically by limiting its availability and encouraging the brain to rely on other substrates for energy such as ketones, is a strategy that has been proven to control certain seizure disorders, including treatment-intractable epilepsy in children [24]. In individuals suffering from both diabetes mellitus and a seizure disorder, improved glycemic control have been associated with a reduction in seizure activity even when antiepileptic drugs were discontinued [25]. In murine research, reduced glucose utilization in the form of long-term calorie restriction has been shown to confer seizure protection in epileptic mice [25, 26]. Nonetheless the relationship between glucose availability and seizures is a complex one, as low glucose availability can also precipitate seizures rather than inhibit it. This is an often-feared adverse effect of antidiabetic drugs such as insulin and sulfonylureas [27].

The interplay between glucose transport, availability, and seizures led us to ponder whether medications that block glucose transport, such as SGLT2 inhibitors, can alter seizure activity. At the time in which this study was initiated, there were limited published studies that investigated the effects of SGLT inhibition on epilepsy. In one study published in 2016, Melo et al. observed [12] that phlorizin, a specific SGLT inhibitor, increased the severity of limbic seizures induced by pilocarpine during status epilepticus and also worsened the severity of neurodegeneration in the hippocampus at 24 h after status epilepticus in mice. The results of this study appeared to contradict a study we conducted, in which phlorizin demonstrated anticonvulsant effects in a dose-dependent manner. Given these early conflicting results, we were motivated to do this study to further investigate the potential effects of SGLT inhibition on seizure activity. Instead of doing another study on phlorizin, we selected dapagliflozin as the study drug since, as far as we know, the effects of SGLT2 inhibitors on seizure activity remained unknown, whether in rats or other mammals [12], and in addition dapagliflozin is becoming widely use around the world as a leading diabetic drug.

There is emerging evidence that SGLTs may play a role in seizure persistence, and that inhibition of SGLTs may serve to stabilize neurons that are at risk of generating or propagating epileptic discharges. There is evidence that SGLT activity is increased in areas of the brain that are actively seizing, to the degree that we can pinpoint the focus of a seizure by tracing radioactive emissions generated from methyl alpha-D-[U-14C] glucopyranoside, an isotope-labeled non–metabolizable substrate of SGLT that tends to accumulate in seizing neurons [23]. Co–administration of penicillin with methyl alpha-D-[U-14C] glucopyranoside can promote further uptake of the radiolabeled isotope in affected neurons, especially in the frontal cortex. Physiologically, SGLTs generate inward currents as they transport glucose into the cytoplasm, as sodium is also transported across the cellular membrane. The concomitant movement of this charged ion can result in increased excitability across the cellular membrane, alongside potential depolarization [8]. While not entirely understood, this may be one mechanism in which SGLTs can accentuate seizure activity. Another potential mechanism is that SGLT inhibitors may result in decreased glucose utilization in the brain, which to a certain extent can reduce seizure activity. In light of these potential mechanisms, inhibition of SGLTs may therefore inhibit seizure activity, and thus provide a putative explanation for our observations in this study.

Another potential therapeutic benefit of using SGLTs to inhibit seizures is that enhanced cerebral SGLT function has also been associated with the development of ischemic neuronal damage [28–30]. The mechanisms that result in a failure of neurons to compensate during this period of high energy demand remains unclear [31].

## Conclusions

In summary, here we demonstrate that inhibition of SGLT2 by dapagliflozin reduces seizure activity in rats stimulated with PTZ with a mean absolute decrease in EEG spike wave percentage of 54.9% (from 75.3 to 20.4%), an improvement in mean Racine’s stages scores, and an increase in time to first myoclonic jerk. These metrics all reached statistical significance with *p*-values of < 0.0001. Higher doses of dapagliflozin (150 mg/kg instead of 75 mg/kg) resulted in a trend towards lesser seizure activity. Inhibition of SGLTs via dapagliflozin and other agents may decrease neuronal glucose consumption and reduce neuronal membrane excitability as well as depolarization events, both of which may be protective against seizures. However, exact mechanisms remains unclear. Our findings add to the body of evidence that SGLTs, and in particular SGLT2, may be involved in the pathogenesis of epilepsy and that SGLT2 inhibitors such as dapagliflozin can be utilized as antiepileptic drugs.

## Abbreviations

°C: Degrees Celsius
AED: Antiepileptic drug
ANOVA: Analysis of variance
BBB: Blood-brain barrier
CNS: Central nervous system
DGF: Dapagliflozin
DMT2: Diabetes mellitus type 2
EEG: Electroencephalogram
GLUT: Glucose transporter
Hz: Hertz
IP: Intraperitoneally
IV: Intravenous
kg: Kilogram
mg: Milligram
NIH: National Institutes of Health
PTZ: Pentylenetetrazol
RSS: Racine’s scales scores
SD: Standard deviation
SE: Status epilepticus
SGLT: Sodium-glucose linked transporter
SLC5A: Solute carrier family 5A
SWP: Spike wave percentage
TFMJ: Time to first myoclonic jerk
